# LC-HR/MS Analysis of Lipophilic Extracts from *Calendula arvensis* (Vaill.) L. Organs: An Unexplored Source in Cosmeceuticals

**DOI:** 10.3390/molecules27248905

**Published:** 2022-12-14

**Authors:** Claudia Gravina, Marika Fiorentino, Marialuisa Formato, Maria Tommasina Pecoraro, Simona Piccolella, Adriano Stinca, Severina Pacifico, Assunta Esposito

**Affiliations:** Department of Environmental, Biological and Pharmaceutical Sciences and Technologies, University of Campania “Luigi Vanvitelli” Via Vivaldi 43, 81100 Caserta, Italy

**Keywords:** *Calendula arvensis*, asteraceae, UHPLC Q*q*TOF-MS/MS analysis, apolar extract, fatty acid, phosphatides, field marigold-based serum, wild plants, native species

## Abstract

As part of a project aimed at promoting the use of *Calendula arvensis* (Vaill.) L. (field marigold, Asteraceae) phytocomplexes in cosmeceutical formulations, the chemical composition in apolar specialized metabolites is herein elucidated. Furthermore, the screening of the cytotoxicity of the apolar extracts was evaluated in order to underline their safety as functional ingredients for cosmetics. After dissection of *Calendula* organs (florets, fruits, leaves, bracts, stems, and roots), ultrasound-assisted maceration in *n*-hexane as an extracting solvent allowed us to obtain oil-like mixtures, whose chemical composition has been highlighted through a UHPLC-ESI-Q*q*TOF-MS/MS approach. Twenty-nine metabolites were tentatively identified; different compounds, among which the well-known poly-unsaturated fatty acids, and oxylipins and phosphatides were detected for the first time in *Calendula* genus. The screening of the dose-response cytotoxicity of the apolar extracts of *C. arvensis* highlighted the concentration of 10 μg/mL as the most suitable for the formulation of cosmeceutical preparations. Sera enriched with leaf and fruit apolar extracts turned out to have the best activity, suggesting it can be used as a new source in skin care thanks to their higher content in fatty acids.

## 1. Introduction

The growing interest of consumers towards ‘everything natural’, coupled with companies’ awareness of an urgent need for a more natural approach to body care, has been the driving force in the replacement of synthetic ingredients, often responsible for some health-associated risks. In this context, wild medicinal and aromatic plants (MAPs) offer unique opportunities of exploitation, as biotic and abiotic factors can influence the content of active molecules [1].

Since ancient times, skin health and appearance improvement have been closely related around the world with the use of natural substances. The interest in natural plant-based products has never waned, and in recent years it has innovated thanks to the growing demand for products that jointly preserve functionality and naturalness [2]. The ancient cosmetic use of plants, and in particular of their oils, to which conditioning, occlusive, soothing, and moisturizing properties are correlated [3,4], is currently being renewed as a precious remedy for the treatment of dermatological disorders, including inflammation, phototoxicity, psoriasis, and atopic dermatitis [5]. In fact, the awareness that oxidative stress plays an important role in skin aging [6], and that prolonged exposure to UV rays reduces the skin antioxidant capacity [7,8] has led to the research and development of multifunctional products based on plant active ingredients. The latter have been extensively investigated due to their ability to protect the skin from exogenous and endogenous factors, and to rebalance lipid homeostasis altered by dermatosis and aging [9]. Thus, natural plant-based cosmetics are proven to promote antioxidant and protective effects, improving skin appearance and preventing UV ray- and oxidative stress-induced damages [10]. In this regard, fatty acids, carotenoids, and polyphenols play an important role. Fatty acids (FAs) are essential for maintaining the normal structure and function of the skin, as they are its constituents. The stratum corneum contains lipids (11%), including water-impermeable glycolipids, adhesive intercellular lipids (cement), and skin coat-forming lipids (NMF) [11], which are responsible for preserving acidic pH and to be an additional protective barrier [12]. Essential fatty acids (EFAs), such as linoleic acid (LA) and α-linolenic acid (ALA), decrease in the epidermis with age, causing sensitivity and roughness of the skin [13], whereas their supplementation, also through topical application, massively improves the skin barrier [14,15]. In addition to fatty acids, carotenoids play a vital role in promoting skin health. Several studies have shown that especially β-carotene, lycopene, lutein, and astaxanthin exert photoprotective effects, due to their light-absorbing properties and their ROS scavenging activity, as well as by regulating UV light-induced gene expression, modulating stress-dependent signaling, and suppressing cellular and tissue responses [16,17,18,19]. Thus, the combination of polyunsaturated fatty acids and carotenoids is to be considered a valid basic strategy for maintaining skin health, and MAPs offer unique opportunities for the recovery and exploitation of these active compounds [1].

In the Mediterranean basin, the Italian vascular flora is the richest in species [20] and represents a precious source of plant resources with potential bioactive molecules poorly studied. In this study, our attention focused on *Calendula arvensis* (Vaill.) L., known as field marigold, an annual herbaceous Euro-Mediterranean species belonging to the Asteraceae family that recently was deeply investigated for its polyphenol and saponins constituents [21], and new findings are in line with the use of the different organs for diversely recovering antioxidant compounds.

The genus *Calendula* includes several species known since ancient times for their therapeutic properties [22], even if only *Calendula officinalis* L. currently has a great interest in the cosmetic market [23,24]. Flower-based preparations of *C. arvensis* are recommended for external use to maintain skin firmness, to prevent skin inflammation, and to regenerate damaged tissues [25,26], and antioxidant, anticandidal, antifungal, cytotoxic, and antimicrobial activity have been attributed to alcoholic and aqueous extracts [27,28].

Thus, as part of a project aimed at promoting the use of *C. arvensis* phytocomplexes in cosmeceutical formulations, our interest is turned on deepening the chemical composition of the *n*-hexane extracts for the valorization of these oil-like mixtures in topical application strategies. Furthermore, this species is very common in its native distribution range and adaptable to different environmental conditions with promising cultivation possibilities.

Therefore, UHPLC-ESI-Q*q*TOF-MS/MS analyses were carried out to evaluate lipophilic constituents in the extracts from the different organs (florets, bracts, fruits, stems, leaves, and roots), whereas after evaluating their dose-response cytotoxicity towards HaCaT and MCF-10 epithelial cell lines, bioactive sera were prepared and their effect on the cellular viability was assessed.

## 2. Results and Discussion

### 2.1. Lipid Profile of the Different Calendula arvensis Organs

**Figure 1:**
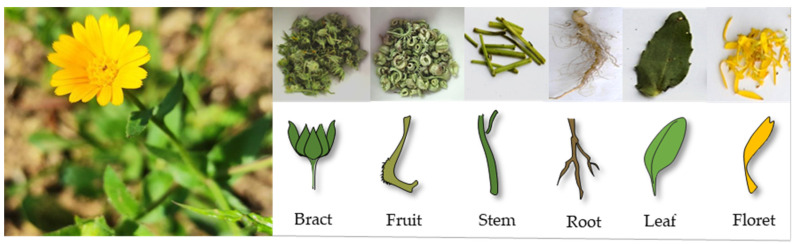
*Calendula arvensis* and its selected studied organs.

The apolar extracts from florets, bracts, fruits, leaves, stems, and roots of *C. arvensis* (Figure 1) were preliminarily investigated for their chemical constituents by means of UHPLC-ESI-Qq-TOF-MS/MS analysis.

Based on ultrasound-assisted extraction, and the use of *n*-hexane as extractant, the diversity in fatty acids of *C. arvensis* organs was unraveled, and twenty-nine metabolites were tentatively identified (Table 1, Table 2, Table 3 and Table 4). Indeed, fatty acids (FAs) were mainly in their free form, whereas glycerol-phosphorylated FAs were detected as minor constituents, together with triterpene aglycones and glycosides. In Table 1, TOF-MS and TOF-MS/MS data of tentatively identified fatty acids are reported.

**Figure 2:**
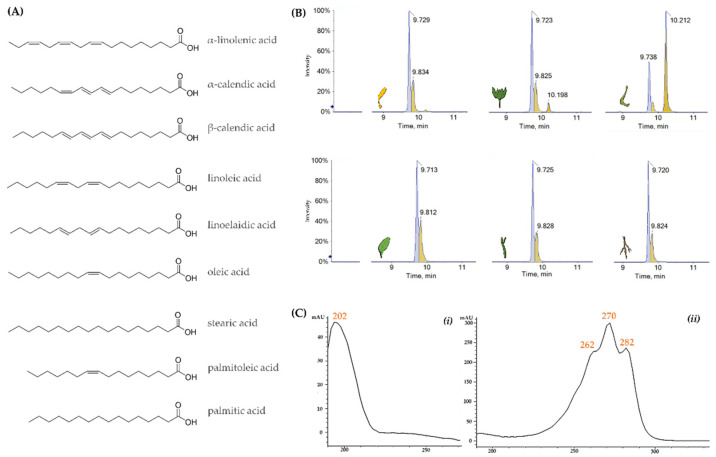
(**A**) Chemical structures of FAs tentatively identified in the extracts of *Calendula arvensis* organs. (**B**) Extracted Ion Chromatograms (XICs) of the ions at *m*/*z* 277.2177 ± 0.05 in the extract of: florets, bracts, fruits, leaves, stems, and roots. (**C**) UV spectra of (**i**) compound **15**, (**ii**) compounds **16** and **17**.

The most abundant compounds were α-linolenic acid (**15**) and its constitutional isomers (**16**, **17**), as well as linoleic acid (**19**). They were previously identified in leaves, seeds, and flowers of a petroleum ether extract of *C. officinalis* L. [29,30]. In particular, the TOF-MS spectra of compounds **15**, **16**, and **17** showed the [M-H]^−^ ion at *m*/*z* 277.2177, according to the molecular formula C_18_H_30_O_2_ (Figure 2). Calendic acid isomers (**16** and **17**), mainly present in fruit organs, were discriminated by α-linolenic acid (**15**) by means of UV-DAD spectra. In fact, due to the occurrence of three conjugated double bonds, their characteristic λ_max_ at 262, 270, and 282 nm were observed [31,32]. These conjugated trienoic acids were likely α-, and β-calendic acids, whose biosynthesis was from linoleic acid by means of a (1,4)-desaturase [33].

Linoleic acid deprotonated molecular ion (**19**) was at *m*/*z* 279.2336, whereas its isomer, likely the linoelaidicic acid (**22**) [30], was also distinguished in florets, leaves, and stems extracts. The [M-H]^−^ ion at *m*/*z* 281.2490 (**28**) was in accordance with the monounsaturated oleic acid, whereas the deprotonated ions at *m*/*z* 253.2177 (**18**) was likely palmitoleic acid. Among saturated FAs, palmitic acid (**24**) with the deprotonated molecular ion at *m*/*z* 255.2331, and stearic acid (**29**; [M-H]^−^ ion at *m*/*z* 283.2643) were tentatively identified in all the investigated extracts. These FAs were previously found as constituents of *C. arvensis* flowers ethyl acetate extract, the latter showing a promising anti-cancer activity against breast cancer cell lines [28]. Based on TOF-MS/MS spectra in negative ion mode, FAs differently shared the [M-H-44]^−^ and [M-H-18]^−^ fragment ions [34,35]. Oxygenated FAs also occurred, and favorably fragmented through β-scission and ene reactions (Table 2). Compounds **5** and **10** with ion [M-H]^−^ at *m*/*z* 295.2284(0), sharing the molecular formula C_18_H_32_O_3_, were tentatively identified as hydroxyoctadecadienoic acids. The different fragmentation pattern of the two compounds allowed us to localize the hydroxyl group at C-13 and C-9, respectively. In particular, the 13-hydroxy derivative (**5**) showed abundant product ions at *m*/*z* 195.1395, which can result from both charge-driven and charge-remote (ene reaction) allylic fragmentation after [1,5]-H sigmatropic shift of the Δ^9,11^ double bonds [36]. The 9-hydroxy derivative was previously found in *C. officinalis* seeds oil [31], with TOF-MS/MS spectra providing the characteristic fragment ion at *m*/*z* 171.1026, due to oxononanoate [37]. An unsaturated keto FA with [M-H]^−^ ion at *m*/*z* 291.1968 (**3**) was tentatively identified as 13-oxo-octadeca-9,11,15-trieonic acid, whereas compounds **7** and **9**, sharing the deprotonated molecular ion at *m*/*z* 293.2122, were likely oxo-octadecadienoic acids, whose recent detection was also in the fruits extract of *C. aegyptaica* Desf [38]. Several mechanisms have been proposed for the biosynthesis of these fatty acids, including formation of an epoxy derivative of linoleic acid as intermediate, oxidation or isomerization of linolenic acid, and formation of a linoleic acid radical by a lipoxygenase-type reaction [32]. Indeed, most of the minor compounds in the investigated extracts were phosphatidic acids (PAs; Table 3). In particular, compounds **1**, **4**, **6**, **8**, and **12** shared the monoacyl glycerol phosphate core, whereas compounds **20**, **23**, and **25**–**27** were diacyl derivatives.

Compounds **1** and **4** with [M-H]^−^ at *m*/*z* 431.2208(07) and molecular formula C_21_H_37_O_7_P, were tentatively identified as isomers of linolenoyl glycerol phosphate (Figure S1A,B). The neutral loss of 154.00 Da was diagnostic of glycerol phosphate, and provided the fragment ion at *m*/*z* 277.2172(71). Furthermore, the ion at *m*/*z* 96.96 was in accordance with phosphate residue. Analogously, compounds **6** and **8** with [M-H]^−^ ion at *m*/*z* 433.2364(8) were linoleoyl glycerol phosphate isomers (Figure S1C,D), and compound **12** with [M-H]^−^ ion at *m*/*z* 409.2366 was likely palmitoyl glycerol phosphate (Figure S1E). The identity of the acyl moiety was through the detection of the ion at *m*/*z* 279.2308(6), corresponding to linoleate, in the TOF-MS/MS spectra of the first two compounds, and of the ion at *m*/*z* 255.2337 (deprotonated palmitic acid) in the spectrum of compound **12**.

The main TOF-MS/MS fragment ions from the diacyl glycerol phosphates (**20**, **23**, and **25**–**27**) arose from the neutral loss of acyl moieties as free fatty acids, and/or of FA-glycerol phosphate moiety (Figure S2). When the monoacyl-glycerol phosphate ion was formed, the nucleophilic attack on the C-1 or the C-2 carbon of the glycerol provided [R_1_CO_2_]^−^ and [R_2_CO_2_]^−^ ions by charge transfer. Thus, compound **20** was tentatively dilinolenoylglycerol phosphate, so much so that its deprotonated molecular ion at *m*/*z* 691.4349 underwent the neutral loss of linolenic acid (−278 Da) to achieve the fragment ion at *m*/*z* 413.2115, which in turn lost an allyl hydrogen phosphate to provide the base peak at *m*/*z* 277.2174 (linolenate). Glycerol phosphate was detected at *m*/*z* 152.9958. Compounds **23** and **25** were likely linolenoyl-palmitoyl glycerol phosphate. The [M-H]^−^ ion at *m*/*z* 671.4672 (**26**) lost 280 Da forming the fragment ion at *m*/*z* 391.2271, likely corresponding to (palmitoyloxy) allyl hydrogen phosphate, or, alternatively, the neutral loss of linoleoyl moiety as a ketene was observed. The ions at *m*/*z* 279.2333 and 255.2335 were in accordance with linoleate and palmitate, respectively. Finally, in compound **27** the phosphatidyl acyl groups are both linoleoyl. Compounds **11** and **21** were tentatively identified as isomers of 16:0/18:2-phosphatidylinositol (Figure S3). The fragmentation pattern resembled that of the glycerol phosphate derivatives described above, and the neutral loss of 162.05 Da was related to inositol moiety. Thus, the deprotonated molecular ion underwent octadecadienoic acid loss to supply the ion at *m*/*z* 553.2815(07), which in turn, after losing dehydrated inositol, generated the palmitoyl-dehydroglycerol phosphate moiety (*m*/*z* 391.2259(71)). Although these metabolites were not previously isolated in the *Calendula* genus, they were recently found in fruits of *Kigelia africana* Benth [39], in seeds of *Camelina sativa* Crantz, and in *Hordeum vulgare* L. roots [40]. Recently, phosphatidic acid was reported to be involved in several cell functions in plants, animals, and microorganisms as a lipid messenger [41]. PAs are associated with various regulatory processes, such as signaling pathways in cell growth, proliferation, and reproduction, as well as responses to hormones and biotic and abiotic stresses [42]. Furthermore, it was shown to exert an antiapoptotic effect on epidermal keratinocytes exposed to oxidative stress caused by hydrogen peroxide [43].

Finally, compound **2**, **13**, and **14** was tentatively identified as triterpenes (Table 4). In particular, compounds **13** and **14** showed the deprotonated molecular ion at *m*/*z* 455.3536(7), and were identified as ursolic acid and oleanolic acid, respectively. GC-MS analysis previously noticed the presence of these compounds in the flower extract of *C. officinalis* L., and literature data showed their ability to exert anti-inflammatory [44], neuroprotective [45], and antimicrobial [46] activities. Compound **2** was tentatively identified as the acetyl derivative of oleanolic acid glucuronide (Figure S4). In fact, in the TOF-MS/MS spectrum the deprotonated molecular ion at *m*/*z* 673.3998 provided the fragment ion at *m*/*z* 631.3903 through the loss of 42 Da (-COCH_3_), which in turn likely underwent water loss to achieve the ion at *m*/*z* 613.3746. Furthermore, the hexuronyl moiety loss (−176 Da) was displayed by the deprotonated molecular ion. The loss of 62 Da from the ion at *m*/*z* 673.3998 to provide the ion at *m*/*z* 569.3872 further confirmed the occurrence of hexuronyl unit [21]. This neutral loss was previously ascribed to simultaneous decarboxylation and dehydration of the saccharidic part [21]. Indeed, *C. arvensis* has been described as source of triterpene saponins based on oleanolic and echinocystic acid. The diversity in the knowledge of these compounds in the *Calendula* genus is herein enhanced by the identification of the compound **2**.

**Figure 3:**
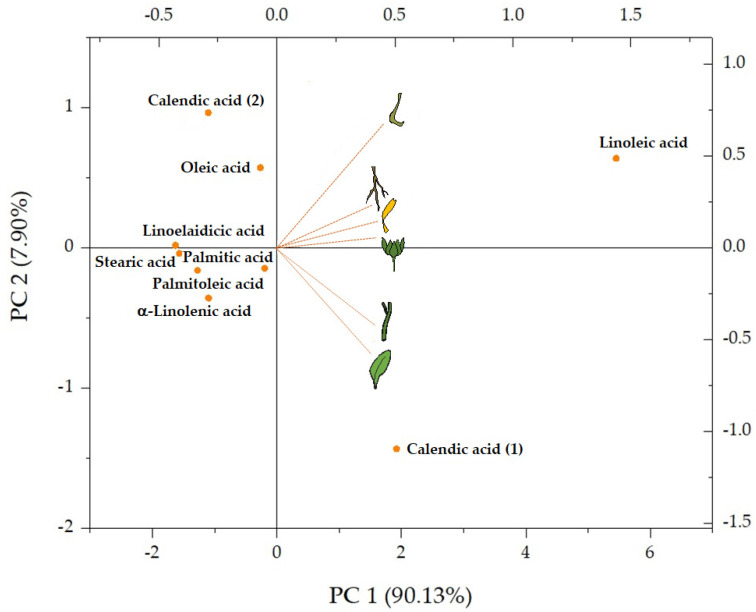
PCA of the free fatty acids tentatively identified in *Calendula arvensis* organs.

The relative quantitation of the tentatively identified fatty acids, oxylipins, phosphatides, and triterpenes highlighted that free fatty acids were the most abundant compounds in all the organs, with the highest amount in leaves and involucral bracts. Principal component analysis (PCA) was carried out considering the relative content of each identified free fatty acid (Figure 3). The two principal components (PC1 and PC2) account for 98.03% of the total variance; the first principal component represents the 90.13% with linoleic acid as the most abundant FAs in all the extracts, being located at the end of the positive score. PC2 underlined leaf and stem richness in calendic acid (**1**), whereas calendic acid (**2**) exceptionally was in fruit extract.

**Figure 4:**
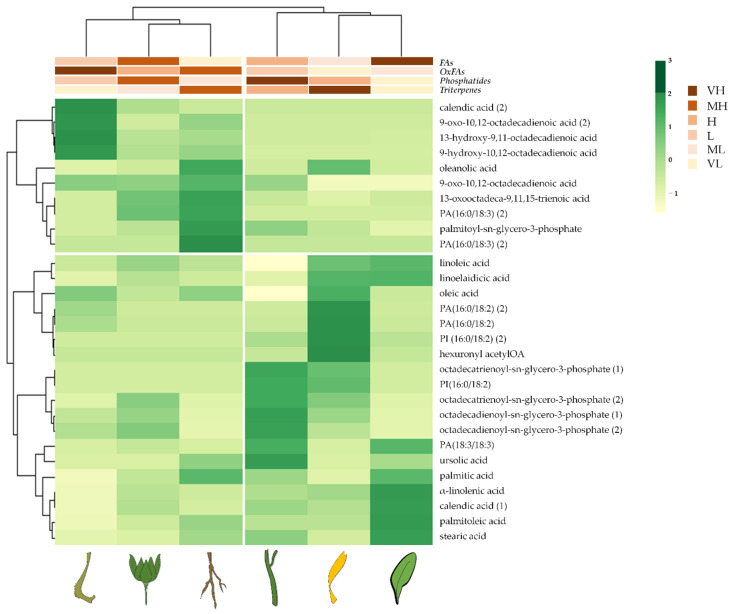
Heatmap of all compounds putatively in *n*-hexane extracts from *Calendula arvensis* organs. On the top, clustering of Fas, oxylipins (OxFAs), phosphatides, and triterpenes are shown. VH: very high; MH: medium high; H: high; L: low; ML: medium low; VL: very low.

The heatmap of the apolar extracts from *C. arvensis* organs showed that cluster segregations occurred based on the relative composition of each investigated organ (Figure 4). In particular, this proves that florets mainly differed for their triterpene content, while leaves were rich in FAs, and seeds in OxFAs. Finally, phospatides were more abundant in stems and leaves than in the other organs.

**Figure 5:**
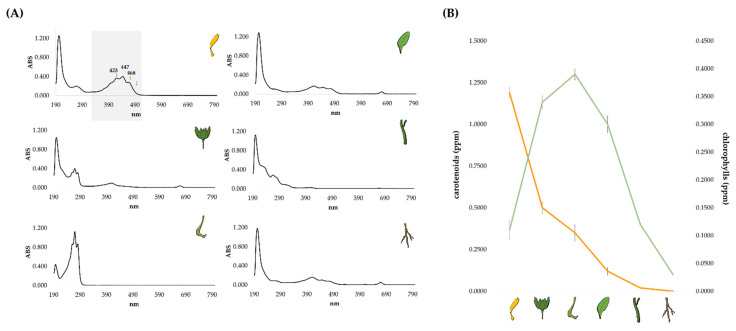
(**A**) UV-Vis spectra of the *n*-hexane extracts from the six organs of *Calendula arvensis*; (**B**) relative content of chlorophylls (ppm; green line) and carotenoids (ppm; orange line) in the investigated organ extracts. Values are the mean ± SD of three independent measurements.

Furthermore, to have a clear picture without deepening the carotenoid compositional detail, the photosynthetic pigments’ content among the different organs was unraveled through UV analysis. The acquired UV-Vis spectra of all the investigated organs (Figure 5A) showed that florets contained an appreciable amount of carotenoid compounds, which are poorly represented also in leaves, stems, and involucral bracts. Florets richness in carotenoids was inversely related to their low chlorophyll content (Figure 5B). Indeed, the UV-Vis spectrum of involucral bracts also detected calendic acid, which further dominated in fruits. According to literature, large amounts of carotenoids have been found in the flowers of the more common *C. officinalis* but with a wide variety of contents, ranging from little to large quantities [47,48,49], which are related to several factors such as plant variety, color of the ligulate and tubular florets, site of cultivation, and harvesting time [47,50,51,52,53].

In the petals of *C. officinalis*, the main carotenoids found were flavoxanthin and auroxanthin [50], while the stems and leaves mostly contained lutein and carotene [48,54]. Carotenoids are biologically active compounds broadly applied in cosmetics, they can act as free radical scavengers and can protect healing wounds [55]. Lutein and its esters are the most abundant carotenoids. This xanthophyll is the basis of supplements useful to reduce the risk of developing age-related macular degeneration (AMD), a degenerative disease, which causes irreversible blindness in the elderly [56]. Only recently an exhaustive investigation was launched on carotenoids and it is necessary to deepen the knowledge of the content of the florets in *C. arvensis*.

### 2.2. Cytotoxicity Screening of Apolar Extracts from Calendula arvensis Organs

HaCaT human keratinocytes and MCF-10 non-tumorigenic epithelial cell lines were used for carrying out the in vitro evaluation of the *C. arvensis* organs’ cytotoxicity through MTT test. HaCaT cells form a reliable in vitro model for studying the functions of keratinocytes, which constitute the 95% of the epidermal cells, as inflammatory/repair response [57] or wound healing effects of plant extracts [58]. MCF-10 cell lines, with a structural similarity to the normal human mammary epithelium, are widely used in cytotoxicity studies as a control to evaluate the safety of different compounds in biomedicine [59,60] and personal care products [61,62]. Thus, both cell lines represent valid models for cytotoxicity tests in dermoprotection studies. After treating cells with increasing doses of *C. arvensis* apolar extracts, the obtained data, analyzed as the average of three replicates, were organized in a matrix (6 organs × 5 concentrations) for each of the two used cell lines. Successively, each data matrix was processed by cluster analysis to explore the degree of values between plant organs and concentration tested. An average linkage agglomeration criterion and Jaccard Index as dissimilarity coefficient were applied.

**Figure 6:**
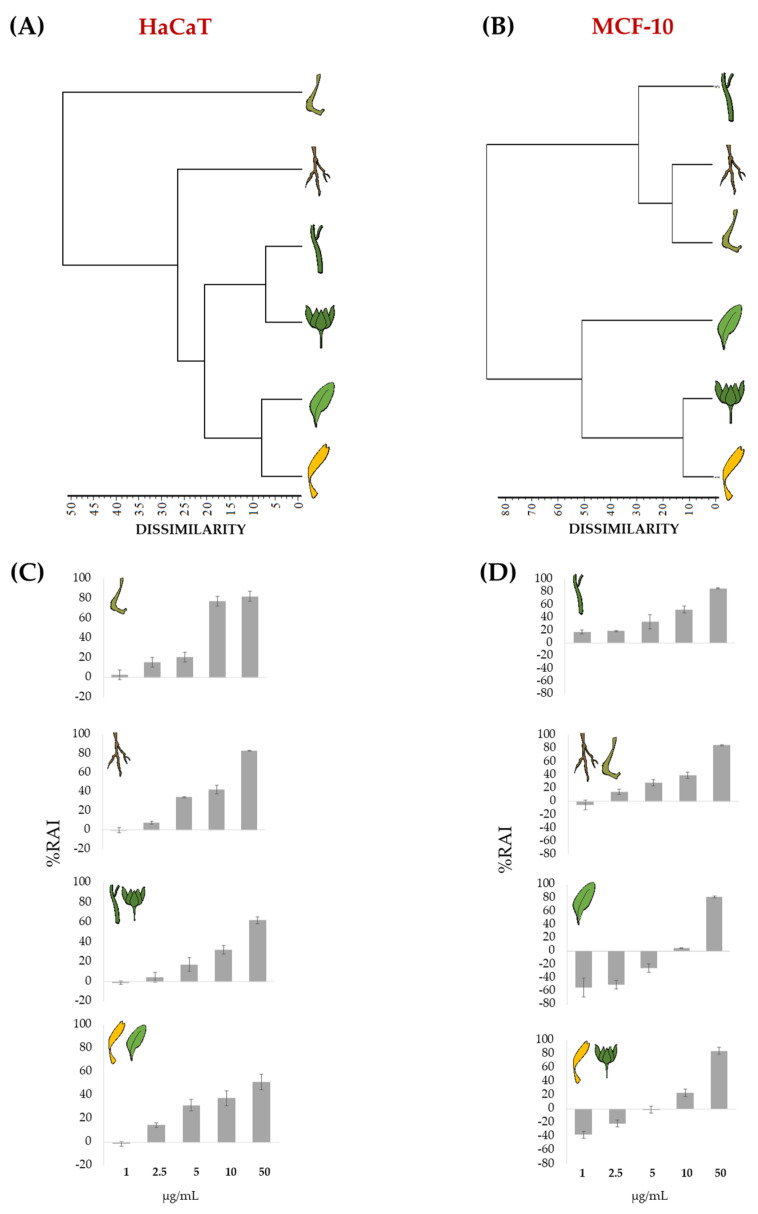
Dendrograms of cytotoxic activity (MTT assay) of apolar extracts of the different organs of *Calendula arvensis* on HaCaT (**A**) and MCF-10 (**B**) cell lines. Redox activity inhibition (%RAI) values of HaCaT (**C**) and MCF-10 (**D**) are ordered as clustered in the dendrograms. Data are expressed as means ± SD of the experiment performed in three replicates.

The obtained dendrograms (Figure 6) clearly showed different clustering patterns of the organs apolar extracts, depending on the cell lines used.

In particular, dendrogram referred to an HaCaT cell line that evidenced two main clusters with a dissimilarity value of 50% (Figure 6A). The first cluster included only the fruit extract, while the second consisted of three subclusters which in turn contained: roots, florets, leaves, bracts, and stems. Cluster analysis on MCF-10 cell line also revealed two main clusters but with 80% of dissimilarity value and each, in turn, composed of two subclusters. The first cluster consisted of the subcluster of leaves, and that grouping florets and bracts. The second cluster also distinguished two subgroups: one containing only stems, and the other with roots and fruits (Figure 6B).

Afterwards, the organ activity data were ordered according to the obtained groups of each dendrogram, respectively (Figure 6C,D), to highlight the cytotoxicity trends in relation to the tested extract concentrations.

With regards to HaCaT cell line, it is clearly observed that the subcluster florets and leaves, as well as that of stems and bracts, are related to the less cytotoxic organs, followed by florets and leaves. All these organs showed a similar behavior in terms of cytotoxicity increase while increasing the concentration. The roots, on the other hand, exhibited a maximum of cytotoxicity at a concentration of 50 μg/mL, whereas the fruit extract showed a very different behavior compared with the other organs, highlighting a strong activity already at the 10 μg/mL tested dose. The data obtained appeared in line with the chemical composition of the organs analyzed. In fact, considering the diversity of florets in triterpenes and phosphatidic acid, it is known that triterpenes exert anti-inflammatory [63], healing [64], and antiproliferative [65] activities. In particular, Ghosh et al. [66] showed that oleanolic acid induced non-appreciable cell death in human keratinocytes compared with cancer cell line, and ursolic acid was involved in reducing the release of pro-inflammatory cytokines NF-κB, IL-6, and TNF-α [67]. On the other hand, phosphatidic acids, which play a key role in intracellular signaling, have been shown to directly activate pro-inflammatory protein kinases [68], and are a source of glycerol and fatty acids. Glycerol exerts beneficial effects on the epidermis by improving the hydration of the stratum corneum, the skin barrier function, and the mechanical properties of the skin, the inhibition of the lipid phase transition of the stratum corneum, the protection from irritating stimuli, the improvement of degradation desmosomal and acceleration of wound healing processes [69]. Furthermore, implementation with *n*-3 and *n*-6 PUFAs showed improvement in psoriasis and atopic dermatitis [70]. Recent evidence showed that PUFAs belonging to *n*-3 class improve inflammatory skin, and the exogenous supplementation of α-linolenic was observed to enhance the skin barrier function thanks to its ability to be incorporated into the phospholipid and triglyceride fractions of the skin. Thus, it can act as modulator of lipid mediators, such as prostaglandins, hydroxy fatty acids, and monoacylglycerols [15].

Oxylipins, which are important constituents in the stem, root, and fruit extracts, are pleiotropic modulators of metabolic and inflammatory responses [71], being regulators differently during cell proliferation, differentiation, and migration [72]. Thus, their higher concentration in the root and fruit extract can explain the higher cytotoxic effect observed. Indeed, in a complex entity such as a plant extract, whose chemistry strongly depends on the extraction method applied and plant organ intrinsic chemical composition [73], the effect can be present through multiple mechanisms. Furthermore, a time- and dose-dependent cytotoxicity can occur, so much so that extracts not exhibiting cytotoxic effects at lower doses are pro-oxidant and cytotoxic when tested at higher doses. Although there are no data in the literature about the relationship between phytochemicals from *C. arvensis* and cytotoxicity profile, the controversial role of *C. officinalis* extracts as a topical agent, also able to be involved in radiodermatitis treatment, was reviewed [74], and extracts obtained through distillation or percolation referred to contain phenolic compounds based on Folin–Ciocalteau assay, were observed as protective in HaCaT cells subjected to hydrogen peroxide insult [75]. Analogously, an aqueous extract from *C. officinalis* was observed to protect HaCaT cells against detrimental effects of oxidative stress-inducing personal care products [76]. The precious diversity in essential fatty acids, and their derivatives in investigated extracts suggested their potential use for counteracting changes in skin lipid components, which can contribute to the onset of different skin diseases. Thus, based on this preliminary cytotoxic screening, the most suitable concentration of the organ extracts to be used in the subsequent phase of the formulation with serum addition was established. In fact, excluding the 50 μg/mL concentration of all the organs, which was highly cytotoxic towards both the tested cell lines, the concentration of 10 μg/mL was chosen as the maximum concentration with the lowest toxicity suitable for formulating cosmeceutical preparations. Although for the chosen concentration, inhibitions of mitochondrial redox activity were observed by the seed extract on HaCaT cells and by the stem extract on the MCF-10 cell line, the concentration was nevertheless adopted for the inclusion of the extract in the formulation.

### 2.3. Cytotoxicity of Cosmeceutical Formulations Based on Calendula arvensis Apolar Extracts

MTT data on HaCaT and MCF-10 cell lines put the basis for the use of the different organs apolar extracts in the development of a cosmeceutical preparation, which can guarantee a beneficial effect on skin. The serum is a highly concentrated product with properties of rapid absorption and the ability to penetrate through the phospholipid bilayer cells. It provides ten times greater richness of biologically active substances than O/W emulsions, thus performing faster and more successfully for skin-related problems [77]. Therefore, the prepared acqueous sera, each one enriched with a different extract (0.2% *p*/*v*), underwent an in vitro screening in order to highlight their cytotoxic potential.

**Figure 7:**
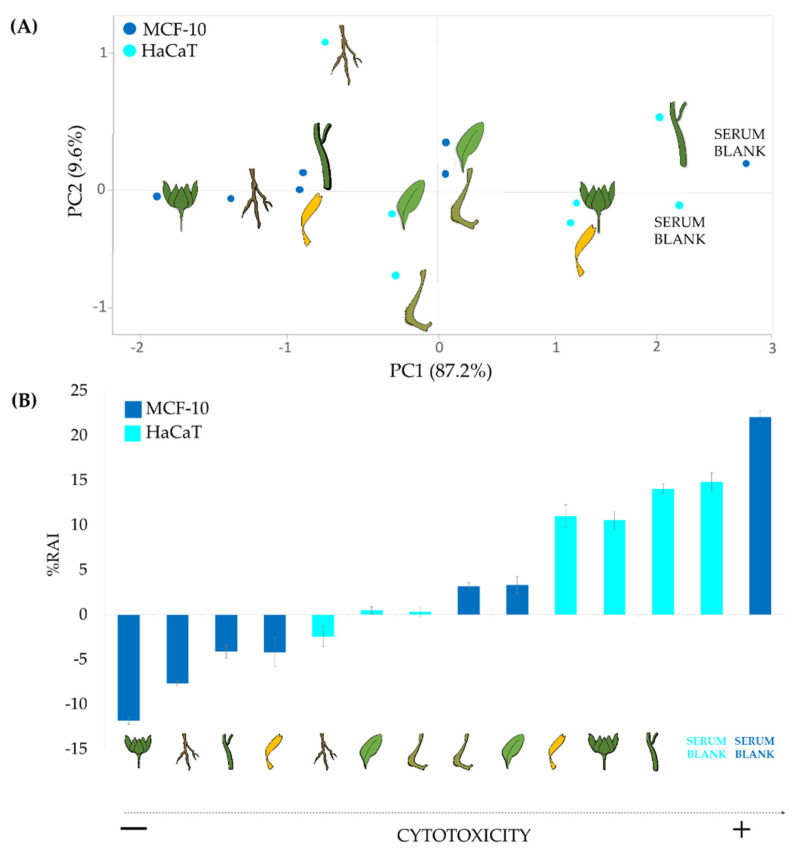
PCA of cytotoxic activity (MTT assay) on HaCaT and MCF-10 cell line of aqueous serum formulated enriched with the apolar extract of *Calendula arvensis* organs (**A**); Redox activity inhibition (%RAI) of each organ ordered along axis 1 as depicted by PCA (**B**). Data are expressed as means ± SD of the experiment performed in three replicates.

The principal component analysis of %RAI of the serum-based apolar extracts makes clear the cytotoxicity gradient along the first axis (PC 1) related to the tested organs and cell lines, with HaCaT being less cytotoxic than MCF-10, and the latter in turn less cytotoxic of the aqueous serum used as blank (Figure 7A). In particular, it was observed that sera enriched with apolar extracts from roots, leaves, and fruits exhibited a similar behavior in both cell lines. Conversely, bracts, flowers, and stems produced greater dissimilarity effects in relation to the target cell line and, in general, a lower cytotoxicity was observed on HaCaT compared with MCF-10.

The RAI percentage of each analyzed organ ordered along axis 1 as depicted by PCA %RAI of each organ showed a low cytotoxic activity (Figure 7B) that does not exceed the value of 20%. In fact, the high inhibition %, equal to 14.07 ± 0.52 was recorded by stem extract, which alone appeared to be more cytotoxic. This trend was in agreement with Balestrin et al. [78] in which the incorporation of *Achyrocline satureioides* (Lam.) DC ethanolic extract into nanoemulsion formulation reduce the cytotoxicity on HaCaT cells compared with free extract treatment [79].

Among all organ extracts-enriched sera tested (Figure 7B), leaves and fruits-included sera showed the best activity with RAI percentage values ranging from very low (0.49 + 0.39 in leaves and 0.31 + 0.53 in fruits) on HaCaT cells, to low (3.32 + 0.98 in leaves and 3.19 + 0.42 in fruits) on MCF-10 cells, thus suggesting that the enriched sera can be considered available and safe thanks to their content in fatty acids and their derivatives. Conversely, flowers and stems displayed a low cytotoxic activity on HaCaT cells, or proliferative activity on MCF-10. This cell-sensitive effect can be due to their triterpene content, which was only partially mitigated by the serum. In fact, previous data underline that ursolic acid was able to induce apoptosis through caspase-3 activation and cell cycle arrest in HaCat cells [80].

To date, there is extensive knowledge of the cosmeceutical preparation of *C. officinalis*, which has 14 INCI designations in the European list of cosmetic ingredients [81]. An aqueous formulation with bio-soothing functional compounds of *C. officinalis* is commercially available, as well as various other *C. officinalis* preparations, mainly extracts, tinctures, and oils to be incorporated into topical formulations aimed at wound healing and to soothe inflamed and damaged skin. *C. officinalis* flower extract is the most used in cosmetic products [82]. Our results on *C. arvensis* are the first to highlight and suggest a potential exploitation of some organs of this species as a new source for skin care, with indications on extract concentration to be used.

As mentioned above, lipids play a crucial role in maintaining normal skin function, and topical application of a formulation enriched in fatty acids extracts can promote this process. Furthermore, free PUFAs have important regulatory effects on the initiation, development, and resolution of inflammation as they can be converted into hundreds of lipid-modulating substances [83]. In this scenario, considering the close link between chemical composition and biological activity, leaves- and fruits-enriched sera represent the most valuable resource for fatty acids topical application being rich in this class of compounds.

## 3. Materials and Methods

### 3.1. Plant Collection and Extraction

*Calendula arvensis* (Vaill) L. was harvested in May 2021 in Roccaromana (latitude 4°16′24.8″ N, longitude 14°12′56.9″ E; 163 m a.s.l), southern Italy. The environmental site characteristics, data on the voucher specimen and organs separation procedure are reported by Fiorentino et al. [21].

**Figure 8:**
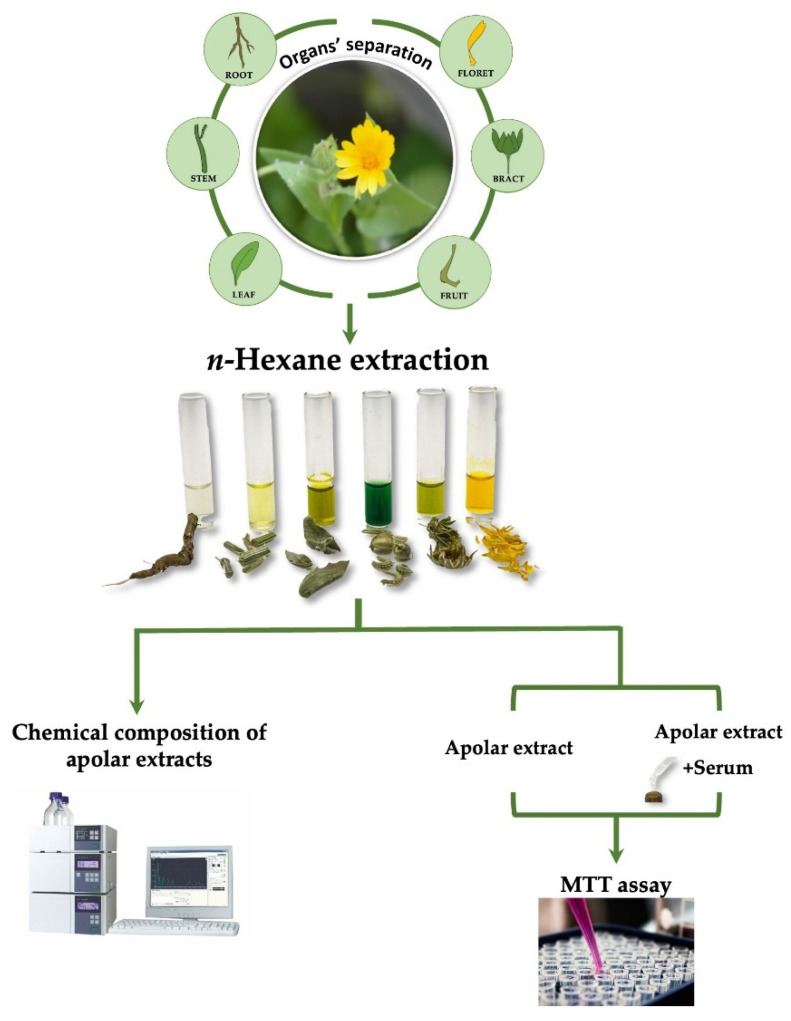
*Calendula arvensis* scheme of methodological steps: organs’ separation and extraction in *n*-hexane followed by chemical composition analysis and cytotoxic assessment of the extract and enriched serum.

The different organs florets, bracts, fruits, stems, leaves, and roots obtained from plant dissection, have been freeze-dried for 3 days using the FTS System Flex-DryTM instrument (SP Scientific, Stone Ridge, NY, USA). The different cryo-dried organs were pulverized by a rotary knife homogenizer (Knife Mill PULVERISETTE 11, Buch & Holm, Herlev, Denmark) and a sample (∼5.0 g) underwent solid–liquid extraction by ultrasound-assisted maceration (UAM; Branson Ultrasonics^TM^ Bransonic^TM^ M3800-E, Danbury, CT, USA) using *n*-hexane as an extracting solvent. Three extraction cycles (30 min each) were performed and at the end of each cycle the sample was filtrated and the extraction solvent was removed using a rotary evaporator (Heidolph Hei-VAP Advanyage, Schwabach, Germany). The apolar extract of each organ was chemically analyzed by UHPLC-ESI-Q*q*TOF-MS/MS (Shimadzu, Tokyo, Japan; AB Sciex, Concord, ON, Canada) and HPLC-UV-DAD analyses (Agilent, Santa Clara, CA, USA). Moreover, the cytotoxicity was assessed on both organs’ extracts and the sera enriched with them (Figure 8).

### 3.2. UHPLC-ESI-QqTOF-MS/MS Analyses

The apolar extracts from the different *C. arvensis* organs were investigated using a NEXERA UHPLC system (Shimadzu, Tokyo, Japan) equipped with a Luna^®^ Omega C-18 column (50 × 2.1 mm i.d., 1.6 μm particle size). The mobile phase was constituted by water (solvent A) and acetonitrile (solvent B), both acidified with formic acid (0.1% *v*/*v*). A linear gradient was used, in which the percentage of solvent B increased as follows: 0–12 min, 5%→32% B; 12–30 min, 32%→75% B; 30–31 min, 75%→95% B; 31–32 min, 95% B. The mobile phase composition was allowed to re-equilibrate for 2 min. The flow rate was set at 0.5 mL/min. High-Resolution Mass Spectrometry (HR-MS) data were obtained by an AB SCIEX Triple TOF^®^ 4600 mass spectrometer (AB Sciex, Concord, ON, Canada), equipped with a DuoSprayTM ion source (AB Sciex, Concord, ON, Canada) operating in the negative ElectroSpray (ESI) mode. A full scan Time-of-Flight (TOF) survey (accumulation time 100 ms, 100–1000 Da) and 8 information-dependent acquisition MS/MS scans (accumulation time 50 ms, 80–850 Da) were acquired using the following parameters: curtain gas 35 psi, nebulizer and heated gases 60 psi, ion spray voltage 4500 V, ion source temperature 600 °C, declustering potential −80 V, and collision energy −40 ± 15 V. The instrument was controlled by Analyst^®^ TF 1.7 software (AB Sciex, Concord, ON, Canada), whereas MS data were processed by PeakView^®^ software version 2.2 (AB Sciex, Concord, ON, Canada). The compounds were identified mainly through the study of their tandem mass spectrometry (TOF-MS/MS; AB Sciex, Concord, ON, Canada) fragmentation patterns, and the comparison with literature data whenever possible.

### 3.3. HPLC-UV-DAD Analyses

To achieve UV-DAD information of fatty acids isomers, separation was also performed by using a 1260 Infinity II LC System (Agilent, Santa Clara, CA, USA) equipped with an Agilent G711A quaternary pump and a WR G7115A diode array detector. The instrument was equipped with Kinetex^®^ PS C-18 (50 × 2.1 mm i.d., 2.6 μm particle size) with a linear gradient in which the percentage of B increases as the following: 0–5 min, 5%→55% B; 5–10 min, 55%→75% B; 10–11% min, 75%→95% B; 11–12 min, and 95% B. The wavelengths were 205, 268, and 282 nm. The flow was 0.4 mL/min.

### 3.4. Chlorophyll and Carotenoids Content

The chlorophyll and carotenoids content of *C. arvensis n*-hexane extracts was spectrophotometrically measured by a Cary 100 Spectrophotometer (Agilent, Santa Clara, CA, USA) against a blank [84]. The chlorophyll and carotenoid content were calculated according to Aladić et al. [85].

### 3.5. Aqueous Serum Formulation

The aqueous serum (100 g) was prepared adding distillated water (70 g) and glycerol (28.5 g) in a glass beaker. The mixture was stirred until the solution was homogenous. Then sodium alginate (1.5 g), used as swelling agent, was added and the solution was mixed by a homogenizer immersion mixer. The mixture was left to rest until it was free of any lamps. All apolar extracts, previously solubilized in pure ethanol, were incorporated in aqueous serum at 0.2% *p*/*w*. The sera, being water-based, are suggested to have a light and rapidly absorbed consistency to penetrate as much as possible into the skin.

### 3.6. Cell Culture and Cytotoxicity Assessment

Human primary keratinocytes cell lines (HaCaT) were cultured in Dulbecco’s Modified Eagle’s Medium (DMEM) supplemented with 10% fetal bovine serum, 50.0 U/mL of penicillin and 100.0 μg/mL of streptomycin, at 37 °C in a humidified atmosphere containing 5% CO_2_. Non-tumorigenic epithelial cell lines (MCF-10) were grown in the same conditions except for the medium, which was FBS-free medium supplemented with 10% Horse Serum.

Cells were seeded in 96-multiwell plates at a density of 1.5 × 10^4^ cells/well. After 24 h, cells were treated with five concentrations of the different organs’ apolar extracts (1.0, 2.5, 5.0, 10, and 50 μg/mL). After 5 h of incubation, cells were treated with 20 mg of MTT (3-(4,5-dimethyl-2-thiazolyl)-2,5-diphenyl-2H-tetrazolium; 0.5 mg/mL), dissolved in the FBS-free culture medium, and allowed to stand for 4 h at 37 °C in a 5% CO_2_ humidified atmosphere. The MTT solution was then removed and 500.0 μL of DMSO were added to dissolve the produced formazan dye. Finally, the absorbance at 570 nm of each well was determined using a Victor3 Perkin Elmer absorbance reader (Perkin Elmer/Wallac, Waltham, MA, USA). Cell viability was expressed as a percentage of mitochondrial redox activity (RAI, %) of the cells treated with the extracts compared with the untreated control, using the following formula [86]:

[(Abs untreated cells) − (Abs treated cells)/(Abs untreated cells)] × 100

Both cell lines seeded in 6-multiwell plates to evaluate the cytotoxicity of serum, used as blank, and sera enriched with the different apolar extracts. The density of HaCaT was 5.0 × 10^5^ cells/well whereas, for MCF-10 was 5.0 × 10^5^ cells/well. The serum concentration used as blank was 36 mg/mL and the formulation enriched was 36 mg/mL plus 1 mg/mL of the apolar extract. After 24 h cells were treated with serum (blank) and serum with all extracts. After 5 h of incubation, MTT (3-(4,5-dimethyl-2-thiazolyl)-2,5-diphenyl-2H-tetrazolium; 0.5 mg/mL) cells’ viability test was carried out as previously described.

### 3.7. Statistical Analysis

A multivariate analysis by ClustVis (https://biit.cs.ut.ee/clustvis/) [87] was applied to explore and clarify quali-quantitative compositive data compounds in each organ. Numerical clustering of MTT assay data was performed to explore the degree of dissimilarity values between plant organs and concentrations tested by using the SYN-TAX software (SYN-TAX 2000, Syntax, Berlin, Germany) [88].

## 4. Conclusions

With the prominent role that fatty acids have in epidermal and metabolic pathways, their analysis is of major interest for the formulation of best products in cosmeceutical research field. The apolar extract obtained with different techniques are usually profiled by means of gas chromatography mass spectrometry (GC-MS). Instead, our work demonstrated that liquid chromatography (LC-MS) represents a pivotal tool for determining the chemical profile of oil-like mixtures, focusing on their EFAs composition. In fact, in *C. arvensis* different classes of compounds not only the well-known PUFAs, but also oxylipins and phosphatides were detected for the first time, widely improving the knowledge of the fatty acid composition of each specific organ of this species and of the whole *Calendula* genus (Table 5). Furthermore, the biological value of FAs confirms that *C. arvensis* offers greater resources to apply in the formulation of a high-functional cosmetic. Specifically, the different organs studied underline that each part of the plant represents a reservoir of specific class of compounds, as PUFAs were the most abundant in leaf and fruit extracts. Lipids in cosmetics are designed to be applied to human skin to preserve and improve its appearance, to form a protective barrier, to preserve from external harmful substances and to promote hydration. PUFAs are commonly used as the main ingredient of personal care products, and the increasing demand of natural-base formulation encourages the deeper investigation of medicinal and aromatic plants (MAPs) as both a traditional and innovative source of bioactive compounds. In this regard, *C. arvensis* proved to be a good candidate to be explored in this field due to its richness and diversity in all the parts of the plant, in different lipophilic compounds clarified by UHPLC-Q*q*TOF- MS/MS analysis. The preliminary cytotoxic screening on two different cells highlighted that the prepared cosmetic, enriched products are safe. Unlike most of the literature data that focused on aerial parts of *Calendula* genus, this work put the basis for intensifying the study of the relationship between chemical composition and bioactivity (wound healing and anti-inflammatory) of each organ that can be applied for specific therapeutic purposes.

## Figures and Tables

**Table 1:** TOF-MS and MS/MS data of FAs tentatively identified in apolar extracts from the different *Calendula arvensis* organs. Peak numbers are based on elution order in the whole reversed-phase chromatograms (RDB: ring and double bond).

Peak	Rt (min)	Tentative Assignment	Formula	[M-H]^−^ Found (*m*/*z*)	[M-H]^−^ Calc. (*m*/*z*)	Error (ppm)	RDB	MS/MS Fragment Ions (*m*/*z*)
15	9.701	α-Linolenic acid	C_18_H_30_O_2_	277.2177	277.2173	1.4	4.0	277.2165 (100); 233.2303
16	9.840	Calendic acid (1)	C_18_H_30_O_2_	277.2177	277.2173	1.4	4.0	277.2173 (100)
17	10.209	Calendic acid (2)	C_18_H_30_O_2_	277.2177	277.2173	1.4	4.0	277.2169 (100)
18	10.232	Palmitoleic acid	C_16_H_30_O_2_	253.2177	253.2173	1.6	2.0	235.1975;153.2051 (100)
19	10.427	Linoleic acid	C_18_H_32_O_2_	279.2336	279.2330	2.7	3.0	279.2201 (100); 261.2237
22	10.679	Linoelaidicic acid	C_18_H_32_O_2_	279.2332	279.2330	0.2	3.0	279.2201 (100)
24	10.872	Palmitic acid	C_16_H_32_O_2_	255.2331	255.2330	1.0	1.0	255.2219 (100); 237.2237
28	11.027	Oleic acid	C_18_H_34_O_2_	281.2490	281.2486	1.4	2.0	281.2482 (100)
29	11.451	Stearic acid	C_18_H_36_O_2_	283.2643	283.2643	0.2	1.0	283.2646 (100); 265.2593

**Table 2:** TOF-MS and MS/MS data of oxygenated FAs tentatively identified in apolar extracts from the different *Calendula arvensis* organs. Peak numbers are based on elution order in the whole reversed-phase chromatograms (RDB: ring and double bond).

Peak	Rt (min)	Tentative Assignment	Formula	[M-H]^−^ Found (*m*/*z*)	[M-H]^−^ Calc. (*m*/*z*)	Error (ppm)	RDB	MS/MS Fragment Ions (*m*/*z*)
3	7.993	13-oxooctadeca-9,11,15-trienoic acid	C_18_H_28_O_3_	291.1968	291.1966	0.8	5.0	291.1974 (100); 247.2065; 223.1685; 195.1400; 111.0821
5	8.185	13-hydroxy-9,11-octadecadienoic acid	C_18_H_32_O_3_	295.2284	295.2279	1.8	3.0	295.2282 (100); 277.2175; 195.1395; 179.1431; 183.1390
7	8.426	9-oxo-10,12-octadecadienoic acid (1)	C_18_H_30_O_3_	293.2122	293.2123	0.3	4.0	249.2215; 185.1188; 96.9607 (100)
9	8.551	9-oxo-10,12-octadecadienoic acid (2)	C_18_H_30_O_3_	293.2122	293.2118	−1.4	4.0	197.1177; 185.1177; 96.9553 (100)
10	8.879	9-hydroxy-10,12-octadecadienoic acid	C_18_H_32_O_3_	295.2280	295.2279	0.4	3.0	277.2154 (100); 171.1026

**Table 3:** TOF-MS and MS/MS data of PAs tentatively identified in apolar extracts from the different *Calendula arvensis* organs. Peak numbers are based on elution order in the whole reversed-phase chromatograms (RDB: ring and double bond).

Peak	Rt (min)	Tentative Assignment	Formula	[M-H]^−^ Found (*m*/*z*)	[M-H]^−^ Calc. (*m*/*z*)	Error (ppm)	RDB	MS/MS Fragment Ions (*m*/*z*)
1	7.823	octadecatrienoyl-*sn*-glycero-3-phosphate (1)	C_21_H_37_O_7_P	431.2208	431.2204	0.9	4.0	431.2208; 277.2172; 152.9964 (100); 96.9696
4	8.011	octadecatrienoyl-*sn*-glycero-3-phosphate (2)	C_21_H_37_O_7_P	431.2207	431.2204	0.7	4.0	431.2206; 277.2171; 152.9964 (100); 96.9696
6	8.424	octadecadienoyl-*sn*-glycero-3-phosphate (1)	C_21_H_39_O_7_P	433.2364	433.2361	0.8	3.0	433.2362; 279.2308; 152.9959 (100)
8	8.520	octadecadienoyl-*sn*-glycero-3-phosphate (2)	C_21_H_39_O_7_P	433.2368	433.2361	1.7	3.0	433.2368; 279.2306; 171.0065; 152.9966 (100)
11	9.041	PI(16:0/18:2) (1)	C_43_H_78_O_13_P	833.5191	833.5186	0.7	5.0	833.5213 (100); 553.2815; 391.2266; 279.2318; 255.2325
12	9.104	palmitoyl-*sn*-glycero-3-phosphate	C_19_H_39_O_7_P	409.2366	409.2361	1.3	1.0	409.2363; 255.2337; 152.9963 (100); 96.9702
20	10.620	PA(18:3/18:3)	C_39_H_65_O_8_P	691.4349	691.4344	0.7	8.0	691.4387; 413.2115; 277.2174 (100); 152.9958
21	10.677	PI(16:0/18:2) (2)	C_43_H_78_O_13_P	833.5192	833.5186	0.8	5.0	833.5224 (100); 553.2807; 391.2259; 255.2325; 223.0004
23	10.766	PA(16:0/18:3) (1)	C_37_H_67_O_8_P	669.4516	669.4501	2.3	5.0	669.4531; 409.2344; 391.2271; 277.2171; 255.2328 (100); 152.9953
25	10.889	PA(16:0/18:3) (2)	C_37_H_67_O_8_P	669.4520	669.4501	2.9	5.0	669.4533; 409.2342; 391.2252; 277.2169; 255.2326 (100); 152.9954
26	11.007	PA(16:0/18:2) (2)	C_37_H_69_O_8_P	671.4672	671.4657	2.2	4.0	671.4704; 391.2271; 279.2333; 255.2335 (100); 152.9962
27	11.007	PA(18:2/18:2)	C_39_H_68_O_8_P	695.4657	695.4672	2.1	6.0	695.4682; 433.2357; 415.2255; 279.2326 (100); 152.9952

**Table 4:** TOF-MS and MS/MS data of triterpenes tentatively identified in apolar extracts from the different *Calendula arvensis* organs. Peak numbers are based on elution order in the whole reversed-phase chromatograms (RDB: ring and double bond).

Peak	Rt (min)	Tentative Assignment	Formula	[M-H]^−^ Calc. (*m*/*z*)	[M-H]^−^ Found (*m*/*z*)	Error (ppm)	RDB	MS/MS Fragment Ions (*m*/*z*)
2	7.992	Acetyl oleanolic acid glucuronide	C_38_H_58_O_10_	673.3957	673.3976	2.8	10.0	673.3998 (100); 631.3903; 569.3872; 497.3671; 483.3508; 455.3545; 113.0245
13	9.493	Ursolic acid	C_30_H_48_O_3_	455.3536	455.3531	1.2	7.0	455.3550 (100)
14	9.665	Oleanolic acid	C_30_H_48_O_3_	455.3537	455.3531	1.4	7.0	455.3551 (100)

**Table 5:** Specialized metabolites of identified *C. arvensis* organs compared with literature. Our data (•), only literature data (○), and both sources (⦿). Co: *C. officinalis*; Csa: *C. suffruticosa* subsp. *algarbiensis*; Ca: *C. arvensis*; Css: *C. suffruticosa* subsp. *suffruticosa*; Cs: *C. stellata*.

	FLORETS	BRACTS	STEMS	ROOTS	FRUITS	LEAVES	AERIAL PARTS	Co	Csa	Ca	Css	Cs
**Oxo Fatty Acids**												
13-hydroxy-9,11-octadecadienoic acid	●	●	●	●	●	●						
9-hydroxy-10,12-octadecadienoic acid		●	●	●	●							
13-oxo-9,11,15-octadecatrienoic acid	●	●	●	●	●	●						
9-oxo-10,12-octadecadienoic acid (1)	●	●	●	●	●							
9-oxo-10,12-octadecadienoic acid (2)	●	●	●	●	●							
**Phosphatidic Acids**												
Octadecatrienoyl-*sn*-glycero-3-phosphate (1)	●		●									
Octadecatrienoyl-*sn*-glycero-3-phosphate (2)	●	●	●									
Octadecadienoyl-*sn*-glycero-3-phosphate (1)	●	●	●	●	●	●						
Octadecadienoyl-*sn*-glycero-3-phosphate (2)	●	●	●	●	●	●						
PI (16:0/18:2) (1)	●		●									
PI (16:0/18:2) (2)	●		●			●						
Palmitoyl-*sn*-glycero-3-phosphate	●	●	●	●	●	●						
PA (18:3/18:3)				●								
PA (16:0/18:3) (1)		●		●								
PA (16:0/18:3) (2)	●		●									
PA (16:0/18:2)	●		●									
PA (18:2/18:2)	●		●									
**Saturated Fatty Acids**												
Palmitic acid	●	●	●	●	⦿	⦿	○	[29,89,90,91,92]	[88,90]	[27,90]	[93]	
Stearic acid	●	●	●	●	⦿	⦿	○	[28,29,89,91,92,94]	[88,90]	[27,90]		
**Mono Unsaturated Fatty Acids**												
Palmitoleic acid	●	●	●	●	⦿	⦿	○	[29,89]		[27]		
Oleic acid	●	●	●	●	⦿	⦿	○	[29,89,91,92]		[27]		
**Poly Unsaturated Fatty Acids**												
α-linolenic acid	●	●	●	●	⦿	⦿	○	[29,90,91,92]	[88,90]	[27,90]	[93]	
Linoleic acid	●	●	●	●	⦿	⦿	○	[28,29,89,90,91,92]	[88,90]	[27]	[93]	
Linoelaidicic acid	●	●		●	⦿	●	○	[29]				
**Conjugated Linoleic Acids**												
Calendic acid (1)	●	●	●	●	●	●						
Calendic acid (2)		●			●	○	○	[28,29]				
**Triterpenes**												
Hexuronyl acetyl OA	●											
Ursolic acid	●	●	●	●	●	○	○	[43,94]				
Oleanolic acid	●	●	●	●		⦿	○	[43,94,95,96]		[97]		

## Data Availability

Not applicable.

## Supplementary Materials

The following supporting information can be downloaded at: https://www.mdpi.com/article/10.3390/molecules27248905/s1, Figure S1: TOF-MS/MS spectrum of the compounds (A) **1**, (B) **4**, (C) **6**, (D) **8**, (E) **12** and proposed fragmentation pathway of monoacyl-PAs tentatively identified in apolar extracts from the different *C. arvensis* organs; Figure S2: TOF-MS/MS spectrum of the compounds (A) **20**, (B) **25**, (C) **27**, and (D) **26**, and proposed fragmentation pathway of diacyl-PAs tentatively identified in apolar extracts from the different *C. arvensis* organs; Figure S3: TOF-MS/MS spectrum of the theoretical [M-H]^−^ ion for compound **11**.

## Sample Availability

Samples of the field marigold oil-like mixtures are available from the authors.
