# Supplementary material for: LC-HR/MS Analysis of Lipophilic Extracts from Calendula arvensis (Vaill.) L. Organs: An Unexplored Source in Cosmeceuticals
Source: Molecules. 2022 Dec 14;27(24):8905. doi: 10.3390/molecules27248905 (PMC9783063; doi:10.3390/molecules27248905)
Supplement: Supplementary file 1 [file molecules-27-08905-s001.zip › molecules-2055861-supplementary.pdf]

molecules

MDPI

Supplementary Materials

LC-HR/MS Analysis of Lipophilic Extracts from *Calendula arvensis* (Vaill.) L. Organs: An Unexplored Source in Cosmeceuticals

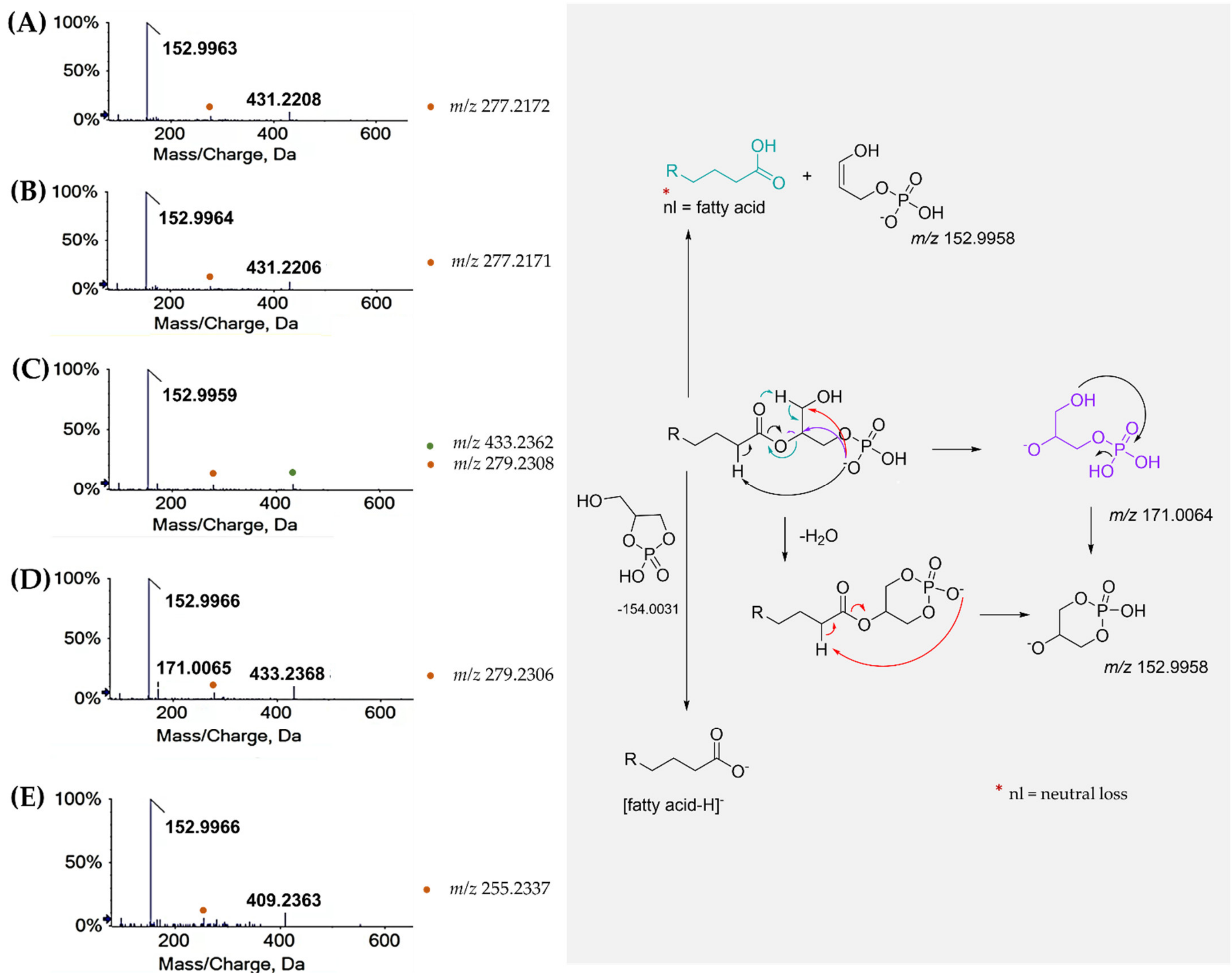

Figure S1. TOF-MS/MS spectrum of the compounds (**A**) **1**, (**B**) **4**, (**C**) **6**, (**D**) **8**, (**E**) **12** and proposed fragmentation pathway of monoacyl-PAs tentatively identified in *C. arvensis* organs' apolar extract (grey box).

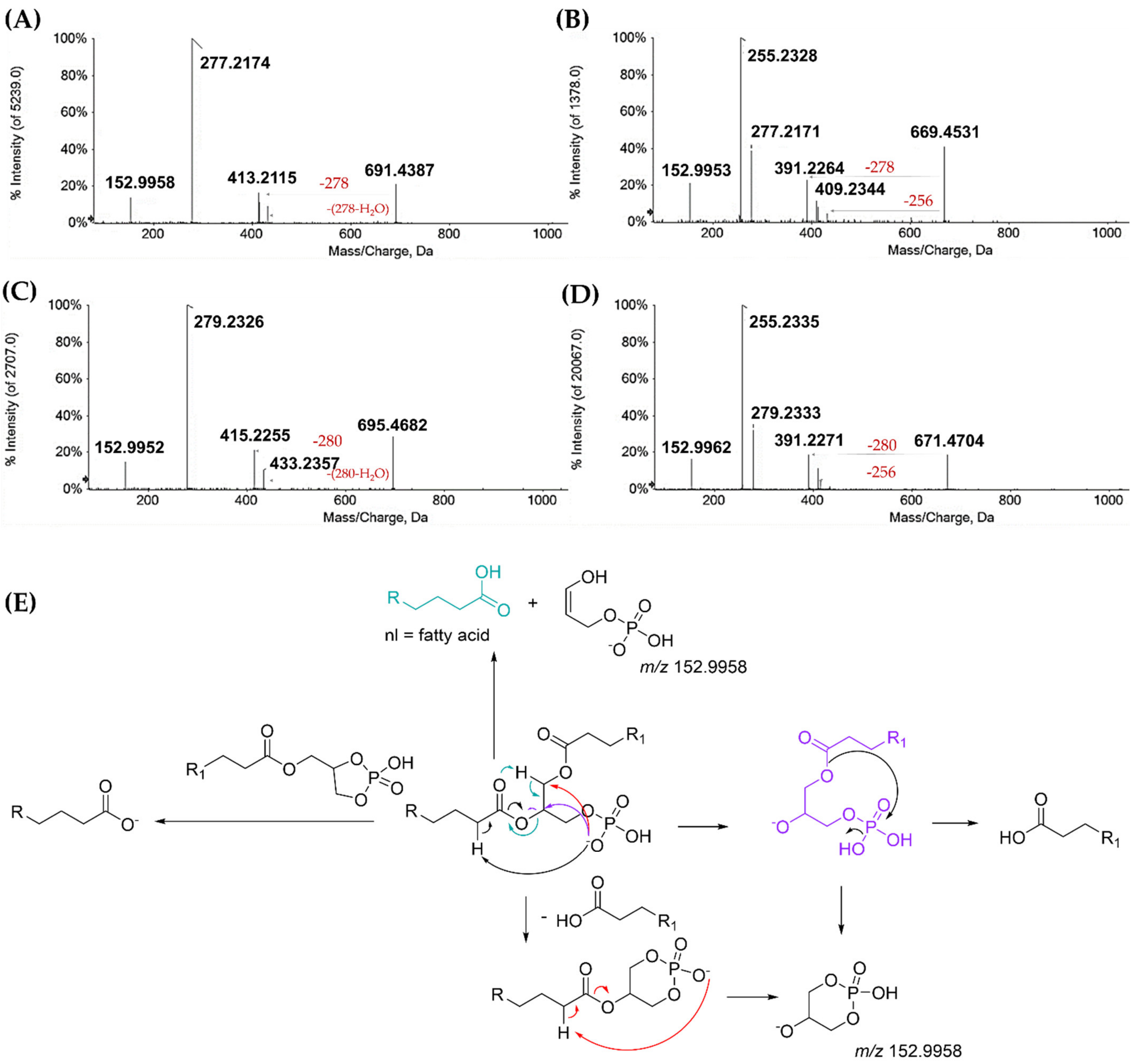

Figure S2. TOF-MS/MS spectrum of the compounds (**A**) **20**, (**B**) **25**, (**C**) **27**, (**D**) **26**, and (**E**) proposed fragmentation pathway of diacyl-PAs tentatively identified in *C. arvensis* organs' apolar extracts.

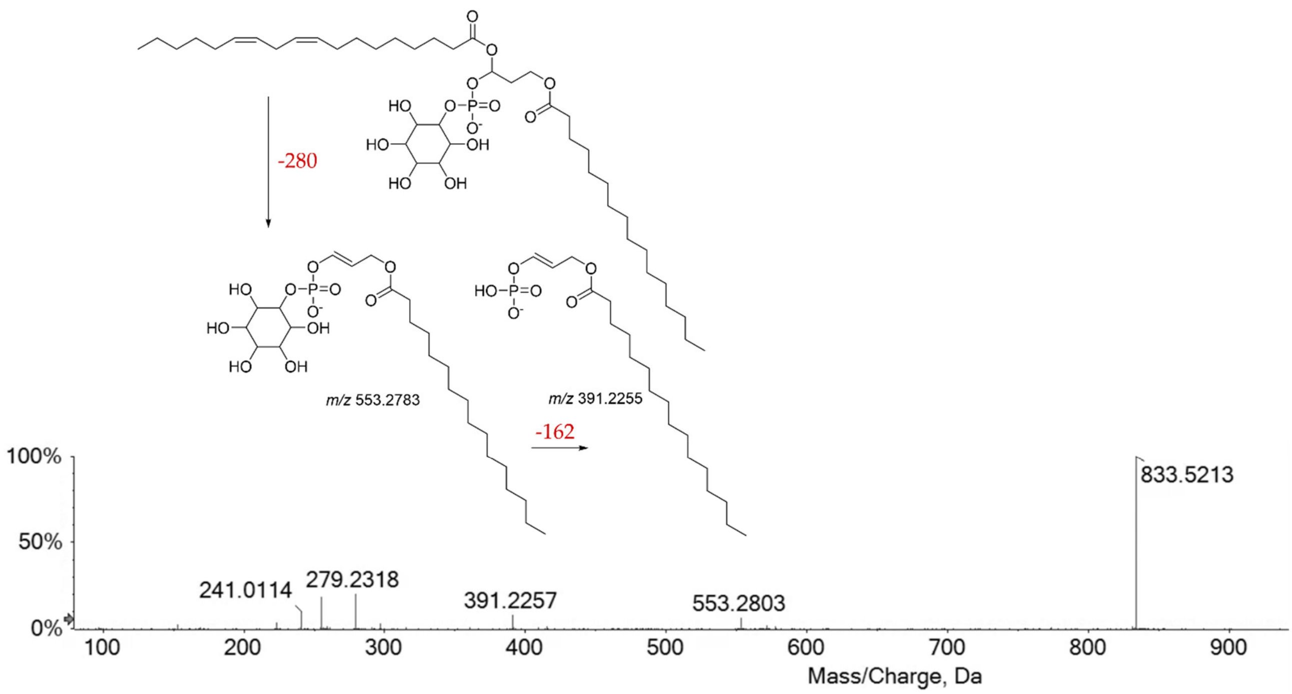

Figure S3. TOF-MS/MS spectrum of the theoretical [M−H]− ion for compound **11**.
